# Conventional Radiology Evaluation of Neonatal Intravascular Devices (NIVDs): A Case Series

**DOI:** 10.3390/diagnostics14020157

**Published:** 2024-01-10

**Authors:** Anna Russo, Vittorio Patanè, Lorenzo Faggioni, Alessandro Pinto, Luigia Fusco, Fabrizio Urraro, Emanuele Neri, Alfonso Reginelli

**Affiliations:** 1 Department of Precision Medicine, University of Campania “L. Vanvitelli”, 80138 Naples, Italy; anna.russo@policliniconapoli.it (A.R.); alessandro.pinto@studenti.unicampania.it (A.P.); luigia.fusco@unicampania.it (L.F.); fabrizio.urraro@unicampania.it (F.U.); alfonso.reginelli@unicampania.it (A.R.); 2 Department of Translational Research and of New Surgical and Medical Technologies, University of Pisa, 56126 Pisa, Italy; lorenzo.faggioni@unipi.it (L.F.); emanuele.neri@unipi.it (E.N.)

**Keywords:** emergency radiology, conventional radiology, pediatric radiology, pediatric emergency radiology, neonatal intensive care unit

## Abstract

Our radiology department conducted an assessment of 300 neonatal radiographs in the neonatal intensive care unit over almost two years. The purpose was to evaluate the correct positioning of intravascular venous catheters. Our case series revealed that out of a total of 95 cases with misplaced devices, 59 were umbilical venous catheters and 36 were peripherally inserted central catheters. However, all of the central venous catheters were found to be properly positioned. Misplacements of neonatal intravascular devices were found to occur more frequently than expected. The scientific literature contains several articles highlighting the potential complications associated with misplaced devices. Our goal is to highlight the potential misplacements and associated complications that radiologists may encounter while reviewing conventional radiology imaging. Based on our experience, which primarily involved placing UVCs and PICCs, we discovered that conventional radiology is the most effective method for assessing proper device placement with the lowest possible radiation exposure. Given the high number of neonatal vascular device placement procedures, it is essential for radiologists to maintain a high level of vigilance and stay updated on the latest developments in this field.

## 1. Introduction

Medical professionals use neonatal intravascular devices (NIVDs) to insert devices into the veins of newborn infants for diagnostic or therapeutic reasons. In recent years, the use of neonatal devices has become more common, especially in pediatric oncology. This is because they are needed for long- to medium-term access to veins for delivering chemotherapy drugs and parenteral nutrition, while also reducing the risks of extravasation and damage to the peripheral vascular endothelium.

In other words, NIVDs are used to provide reliable and long-lasting access to the bloodstream of newborn infants, especially those who need chemotherapy or parenteral nutrition. This is important because it helps to reduce the need for repeated needle sticks and the risk of complications.

NIVDs comprise central venous catheters (CVCs), peripherally inserted central catheters (PICCs), and umbilical venous catheters (UVCs), each with a unique access route. CVCs are frequently inserted through the right jugular or left jugular to deliver large volumes of chemotherapy drugs to oncology patients via a vein with sufficient flow and caliber, while minimizing harm to the peripheral vascular system.

PICCs are typically placed peripherally, at the brachial level, whereas UVCs are inserted into the umbilical venous system. While NIVDs are typically considered safe, there are still potential complications such as venous infections and thrombosis.

Patients with immunosuppressive diseases and those undergoing drug treatments for oncology are at an increased risk of infection. Venous thrombosis, on the other hand, is more prevalent in adult patients with diabetes, COPD, or metastases, which are less common in pediatric patients [1,2].

**Figure 1:**
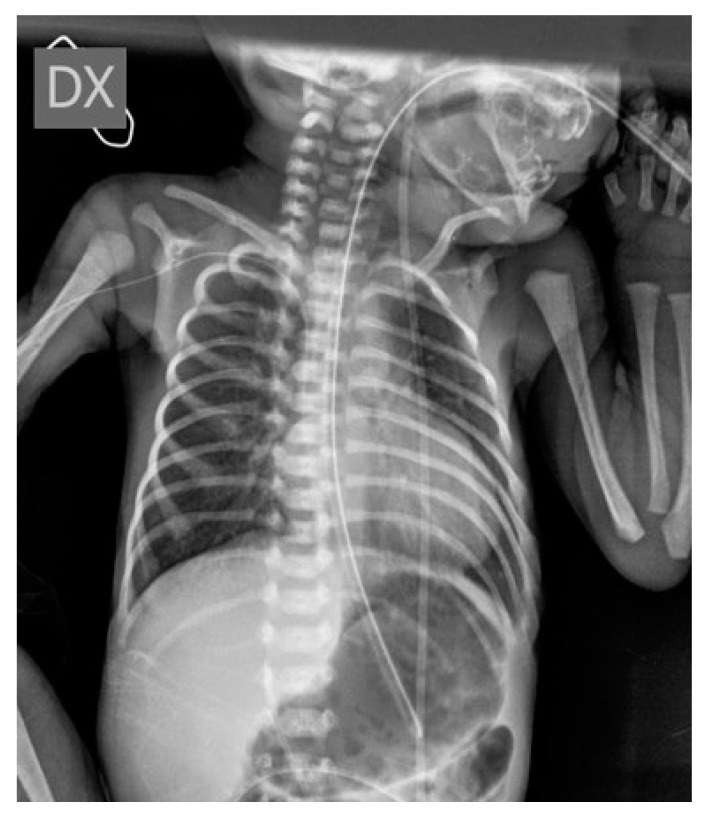
The peripherally inserted central catheter (PICC) of the male infant has been correctly placed, with its tip located at the level of the sixth dorsal vertebra. Nasogastric tube is present and well positioned too.

CVCs are typically inserted from the subclavian vein or internal jugular vein, with a small diameter of about 3 mm. They are radiopaque without a reference strip. The catheter’s path usually travels through the medial end of the clavicle, descends laterally to the right side of the spine, and finally projects into the superior vena cava or the cavoatrial junction. This area is typically located between the fifth and sixth thoracic vertebrae (T5–T6), at least two vertebral bodies below the carina, as shown in Figure 1 when correctly positioned.

**Figure 2:**
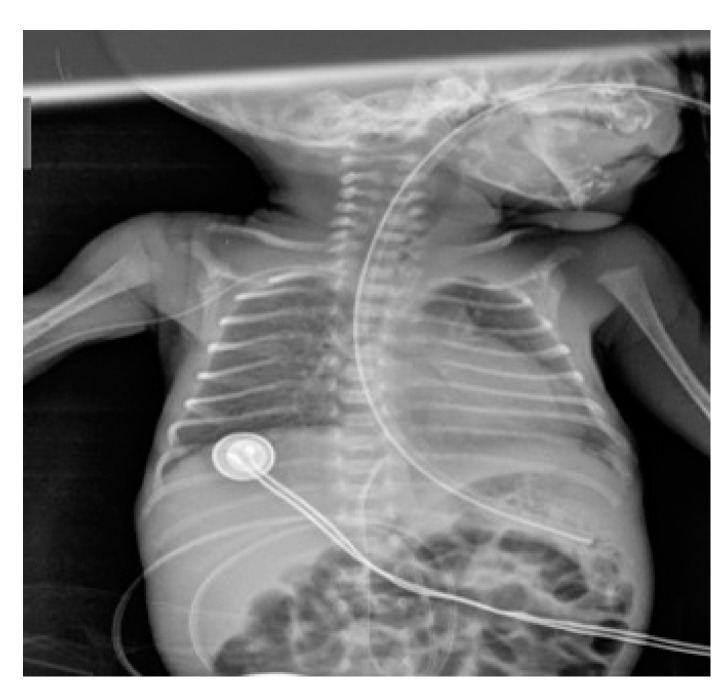
The umbilical venous catheter (UVC) has been correctly positioned in a male newborn, with its distal tip projecting at the level of the 9th dorsal vertebra. The accompanying image also shows that both the peripherally inserted central catheter (PICC) and nasogastric (NG) tube have been appropriately placed.

The misplacement of CVCs can lead to the distal end projecting into the right atrium or internal jugular, which may cause arrhythmias. In some cases, the catheter can also create angled kinks and twists or even perforate the venous vessel. On the other hand, when properly inserted through the brachial vein, PICCs are thin and radiopaque with their distal end projecting at the level of the superior vena cava, usually between the fifth and sixth thoracic vertebrae (as depicted in Figure 2).

Incorrect positioning can elevate the risk of venous thrombosis or dislocation, with pneumothorax (PNX) being a rare complication in about 5% of cases [3,4,5,6,7,8,9].

UVCs are radiopaque and typically inserted through the umbilical vein to provide rapid venous access in the first few hours of neonatal life. However, they are associated with frequent complications, and guidelines from the Centers for Disease Control and the Infusion Therapy Standard of Practice recommend against their long-term use (maximum 14 days) [10,11,12].

**Figure 3:**
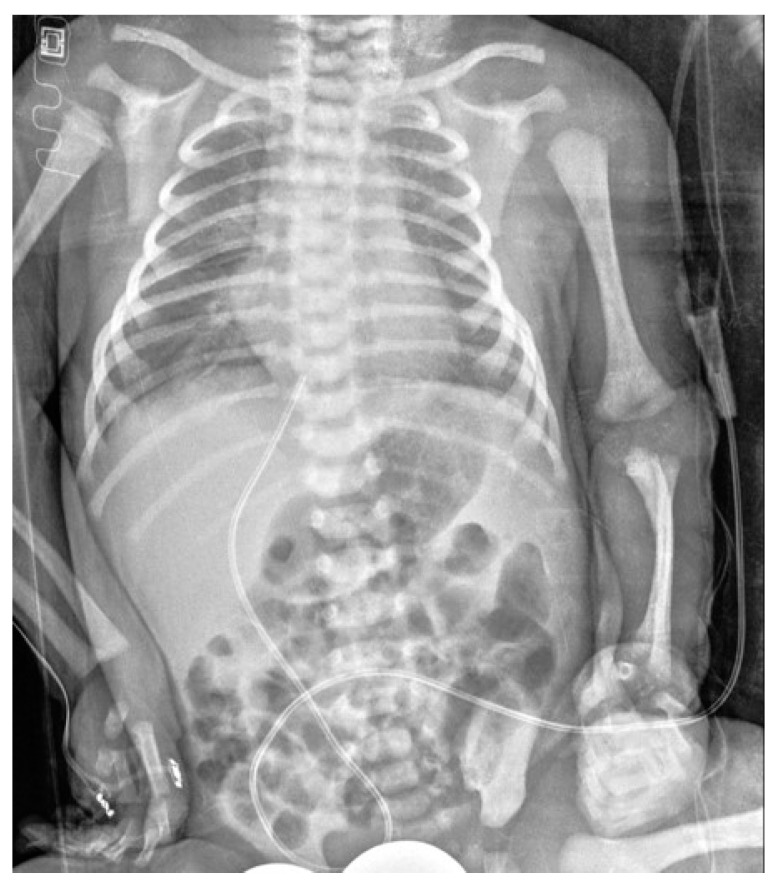
The inferior vena cava cannulation in a female newborn has resulted in a satisfactory position of the distal apex of the UVC at the T9 level.

**Figure 4:**
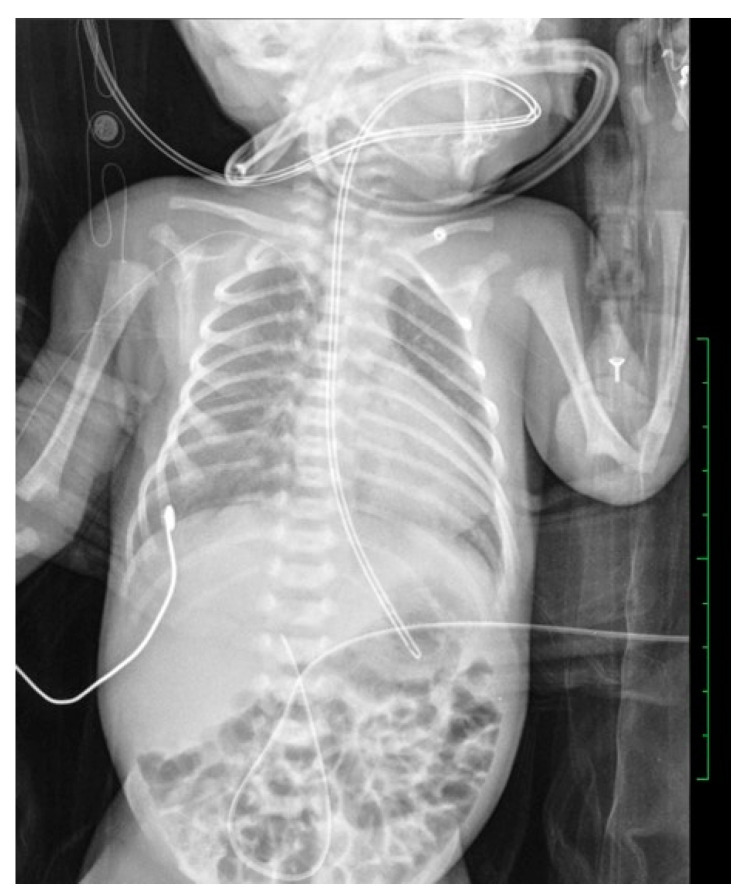
The distal apex of the UVC in a male newborn, born an hour prior, has been inaccurately positioned within the umbilical vein at the level of L3.

UVCs are suitable for emergency access, the infusion of fluids, hyperosmolar solutions, medication administration, central venous pressure (CVP) monitoring, and transfusions. Monolumen and bilumen are the two types of umbilical venous catheters available. Monolumen catheters are suitable for infants who only require infusion therapy (Figure 3), while bilumen catheters are preferred for repeated withdrawals or CVP monitoring (Figure 4).

However, due to their larger surface area, bilumen catheters have a higher risk of thrombosis [13,14,15]. The umbilical vein (UV) in a full-term newborn is approximately 2–3 cm long and has a diameter of 4–5 mm [16]. It courses upwards and slightly to the right from the umbilicus, and joins the left branch of the portal vein after branching out into many large intrahepatic vessels that distribute directly into the liver tissue. Arantius’ duct, a continuation of the umbilical vein, originates from the left branch of the portal vein, opposite the umbilical vein outlet. At birth, the ductus venosus is located between the right and left lobes of the liver, with a length of 20–30 mm and a diameter of 4–5 mm, and joins with the hepatic veins to empty into the inferior vena cava (IVC). The ligation of the umbilical vein after birth causes the exclusion of Arantius’ duct. Subsequently, it transforms into a venous ligament and is anatomically obliterated between the 30th and 90th days of life. The intra-abdominal portion of the umbilical vein also transforms into the round ligament of the liver by the 3rd month of life through a process of obliteration. Proper placement of the umbilical venous catheter involves passing through the umbilical vein to the left portal vein, then through the ductus venosus, past the hepatic veins, and finally into the inferior vena cava. This procedure requires the assistance of a dedicated medical and nursing specialist team. It is crucial to promptly detect and correct any misplacement of umbilical venous catheters to avoid severe complications.

Some of the common misplacements include:

- An insertion too low in the umbilical vein, which can impede appropriate drug delivery;
- An insertion at the level of the right or left portal venous system, superior mesenteric or splenic vein, which can increase the risk of venous thrombosis, portal perforation resulting in liver abscesses and hemorrhage, necrotizing enterocolitis (NEC), and portal hypertension;
- An insertion too high in the right or left atrium (depending on the patency of the foramen ovale), which can lead to cardiac arrhythmias, pericardial effusions with cardiac tamponade, pleural effusion, and endocavitary thrombosis.

It is crucial to have a dedicated medical and nursing team to ensure the proper placement of the catheter and to monitor for any signs of misplacement or complications. Based on the scientific literature, the optimal position for an umbilical venous catheter is at the junction of the inferior vena cava and the right atrium, usually at the T8–T9 level. However, some researchers, like Gibson et al., suggest a position at T9–T10. It is advisable to place the catheter far away from the origin of the hepatic vessels and portal vein to reduce the risk of complications and adverse events. Drug infusion minimizes the chances of complications, as supported by various studies [10,17,18,19,20,21,22]

## 2. Materials and Methods

A retrospective analysis was conducted on 300 chest and abdomen radiographs of neonates, of which 108 received NIVDs at the neonatal intensive care unit (NICU) department of University Hospital “Luigi Vanvitelli” in Naples, Italy, between December 2020 and December 2022. The radiographs were captured using portable General Medica Merate-TMS320 devices (GENERAL MEDICAL MERATE, Seriate, Italy) within 24 h of catheter placement, and in a single antero-posterior (AP) projection. The voltage range used was 50 to 65 kVp, and the tube load varied between 0.5 and 3 mAs to minimize exposure time. The study included 210 Italian neonates and 113 neonates of non-EU nationality, primarily from Pakistan and India, who were between two hours and two days old. Written informed consent was obtained from the minors’ legal guardians for the publication of any potentially identifiable images or data included in this manuscript. All images were anonymized before the publication.

## 3. Results

In our case series, we observed multiple instances of venous catheters being misplaced. We focused primarily on UVCs due to the young age of the patients, but also considered PICCs and CVCs in 120 neonatal patients to a lesser extent. A total of 95 cases were identified where devices were mispositioned. Of these, 59 UVCs were improperly positioned, with 28 located in the portal position, 9 in the left ventricular position, 4 in the inferior mesenteric position, 10 in the umbilical position, 5 in the superior mesenteric position, and 3 in the splenic position. Additionally, we found 36 PICCs that were improperly positioned, with 18 located in the left atrial position, 13 in the jugular position, and 5 in the subclavian position. However, all central venous catheters (CVCs) were positioned correctly. We did not observe any significant adverse events or complications in our case series.

## 4. Discussion

**Figure 5:**
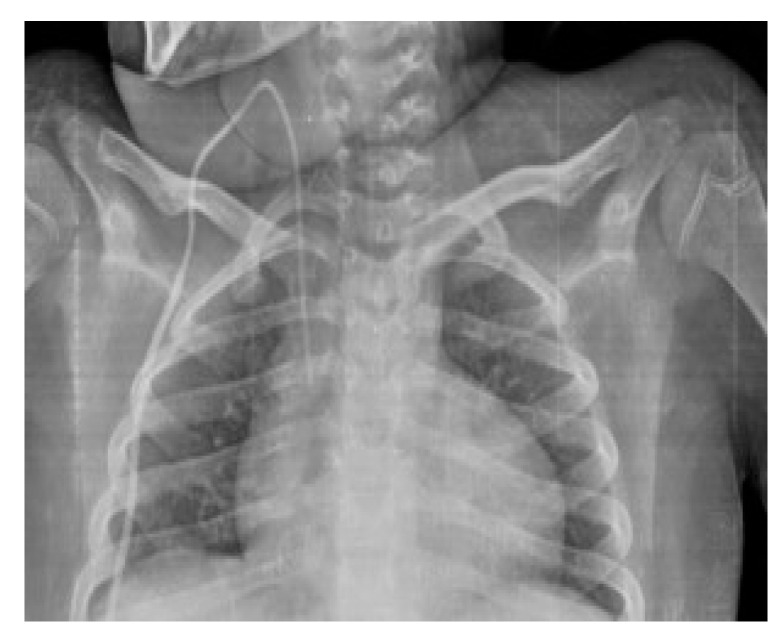
The distal apex of CVC in a male, 8 years old, has been appropriately positioned in the right atrium at the level of the posterior arch of the fourth rib and between the 5th and the 6th thoracic vertebra.

To provide guidelines for the proper positioning of venous catheters, we conducted a literature review that focused on UVCs commonly used in our NICU service. Our case series only included pediatric and newborn patients, specifically those in the neonatal period (0–4 weeks of age) [23]. The ideal placement of the distal apex of UVCs is suggested to be at the level of the seventh to eighth thoracic vertebrae (Figure 3), while for CVCs (Figure 5) and PICCs, it is recommended to position them between the fifth/sixth thoracic vertebrae.

**Figure 6:**
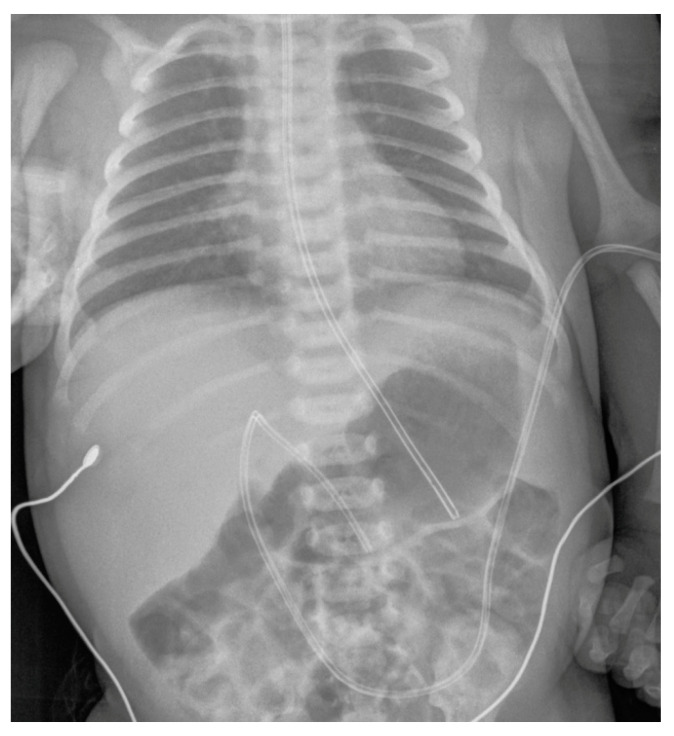
The placement of UVC is inaccurate in a male infant born five hours prior, with the distal apex of CVO positioned at the level of L4 in the inferior mesenteric vein.

These locations promote optimal intravascular drug delivery while reducing the risk of adverse events, as supported by the scientific literature and clinical experience [24]. Our literature review discovered that the most frequent misplacements for UVCs occurred either too low at the pre-hepatic level (Figure 6) or too high at the superior vena cava level, with less common occurrences in intrahepatic, portal vein, or intracardiac locations.

Additionally, we noted device loops in some cases (Figure 6). Our focus was on providing guidelines for the proper positioning of UVCs that are frequently utilized in our NICU service, especially in 151 pediatric patients, particularly those in the neonatal period (0–4 weeks of age) [25].

**Figure 7:**
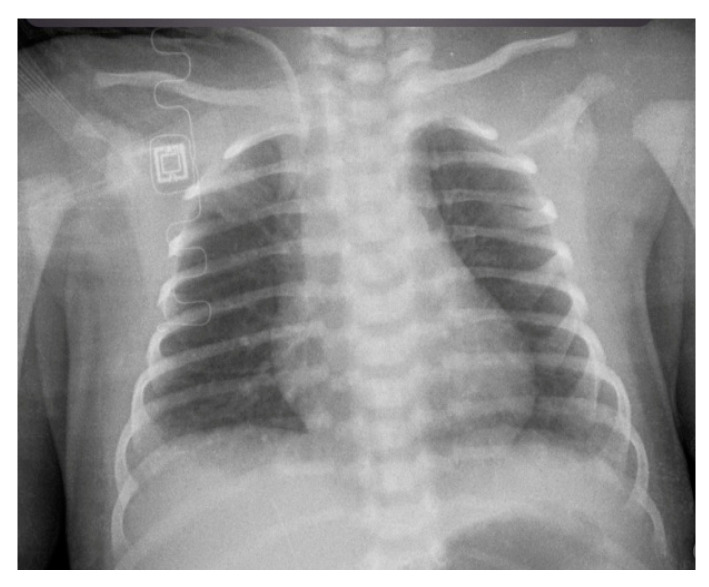
The distal end of the PICC in a male newborn has been inaccurately placed within the internal jugular vein at the level of the first rib’s posterior arch.

Misplacement of CVCs and PICCs was most commonly observed when the catheter was either too close to the subclavian vein (Figure 7) or too far into the right atrium, with few other misplacements seen.

These results are in agreement with those reported in the scientific literature on the frequency and presentation of intravascular device misplacements. Although there are numerous studies in the literature on complications related to PICC placement, limited research is available on the proper site for the placement of intravascular devices, particularly umbilical venous catheters. The use of traditional radiology and post-catheterization follow-up has decreased the risk of complications and misplacements. Radiologists are critical in identifying unsuitable catheter positions, facilitating the repositioning or removal of the catheter when necessary. Radiographic monitoring should be conducted within 24 to 48 h after catheter insertion, as this period is deemed crucial [1,26,27].

Thomas M Vesely presents a different approach to the placement of CVCs in his publication in the Journal of Vascular and Interventional Radiology [23]. He highlights ongoing debates surrounding the optimal positioning of catheter tips, which should take into account the intended use of the catheter. For instance, a distal placement of the catheter tip in the SVC, either at or slightly above the carina level, is generally acceptable for short-term uses such as fluid administration or central venous pressure monitoring. For catheters intended for long-term chemotherapy, they may be positioned lower down, closer to the cavoatrial junction. Lastly, catheters used for hemodialysis may be placed at the cavoatrial junction or even within the right atrium itself [23].

If the considerations mentioned earlier are not taken into account, the cavoatrial site remains the site with the lowest risk. This site does not require differentiating the catheter’s intended uses and has more scientific evidence supporting its use [8,28,29,30].

According to the scientific literature, the appropriate site for UVCs is the junction where the inferior vena cava meets the right atrium [2,17,18,19,20,21,22,31].

The misplacement of UVCs can result in various complications, with the most common misplacements being either too high, projecting at the level of the right atrium, or too low, in the portal vein, hepatic vein, or ductus venosus. In some cases, the distal end of the catheter may project below the liver into the umbilical vein.

If the catheter is too close to the portal system, there is an increased risk of venous thrombosis, leading to portal hypertension. Hemoperitoneum or hepatic hematoma can also occur if a hepatic or portal vessel is perforated. Complications such as infarction, liver necrosis, or necrotizing enterocolitis may arise if hypertonic fluid extravasates into the liver [1,2,21].

Placement of the catheter tip at the right atrial level may lead to arrhythmias, lacerations, or cardiac tamponade, while placement at the level of the pulmonary artery may exponentially increase the risk of hemothorax or gas embolism [32,33].

If the distal apex of the catheter is projected to an unknown vascular site, an investigation for potential risks is necessary [34]. The radiologist should identify and address the misplacement by repositioning or removing the catheter in all cases. According to the scientific literature, UVCs should be used for a maximum of half a week, and then peripheral access catheters may be considered. The duration of use may vary between 7 and 14 days based on the experience of the referral center, but shorter use of the catheter appears to have lower risks of adverse events [11,13,14,35].

According to a meta-analysis conducted by Gibson and colleagues, which examined 14,226 venous catheters utilized between 2010 and 2020, adverse reactions related to UVCs were relatively frequent. Approximately 13.4% of the catheters examined were associated with minor or serious adverse events; however, timely recognition and intervention reduced the likelihood of fatal events, which were infrequent. Malposition was the leading cause of adverse events, accounting for 41.7% of cases, followed by catheter tip migration (36.7%), septicemia and infection (6.9%), and thrombosis (6.5%). Other complications, such as arrhythmias, hepatic and cardiac damage, catheter extravasation, and occlusion, were less common, occurring at a frequency of around 1.5% [21].

The radiologist’s report should include precise details of the catheter’s distal apex location, utilizing significant anatomical or vertebral landmarks. This information is crucial for clinicians to fully comprehend the radiogram evaluation. In addition to catheter placement, any complications noticed during chest or abdominal X-rays should be documented in the report. For example, CVCs and PICCs should be examined for pneumothorax, while pulmonary opacities on chest X-rays can be indicative of lung infections [36]. There are other complications that are well-documented such as pleural effusion or hydrothorax [37]. These can be caused by the perforation of a central vein during or after catheter insertion, obstruction of the thoracic duct outlet by the catheter tip or thrombus, erosion of vessels due to mechanical and chemical irritation, infusion of hyperosmolar solutions that may damage the vessel without direct perforation by the catheter, and thrombosis of the superior vena cava, which can result in chylothorax. Additionally, catheter ruptures, intestinal occlusions, and perforations caused by necrotizing enterocolitis (resulting from the occlusion of mesenteric vessels) have been reported. Lastly, hepatic vessel rupture may occur, which can cause extravasation into the peritoneal cavity, resulting in ascites and/or hemoperitoneum. The use of a chest X-ray can provide indications of thrombosed embolisms, which may be identified through signs like Westermark’s sign, Hampton’s sign, or pleural effusion [1,2,31,38,39].

In addition to addressing the clinical question, it is crucial to report any other potential findings. It is important to remain vigilant and knowledgeable about venous catheter placement to ensure that medical staff are aware of the associated risks.

## 5. Conclusions

The primary method for evaluating the misplacement of intravascular devices is through X-rays. This method is widely accessible, involves minimal radiation exposure, and can be performed portably. Typically, X-rays are conducted within 24 to 48 h after the device is placed to identify potential complications and misplacements. Due to the large number of intravascular catheters used annually, particularly in pediatric and neonatal settings, and the growing dependence on these devices in new therapies, it is essential to maintain high levels of medical and nursing education regarding the associated risks [40]. Radiologists play a pivotal role in determining the catheter’s positioning and identifying related complications, and it is critical to provide unambiguous and accurate reports on the catheter apex location to enable clinicians to choose the best procedural and therapeutic options.

## Data Availability

Data supporting the reported results can be requested from the corresponding author.
